# Evidence on the Effectiveness of Public Policies for Physical Activity Promotion in the Early Childcare Education and Care Setting: A Systematic Review

**DOI:** 10.1111/cch.70078

**Published:** 2025-04-14

**Authors:** Maike Till, Kevin Volf, Clara Tristram, Stefanie Do, Peter Gelius, Antje Hebestreit, Sylke Oberwöhrmann, Sven Messing

**Affiliations:** ^1^ Department of Sport Science and Sport Friedrich‐Alexander‐Universität Erlangen‐Nürnberg Erlangen Germany; ^2^ Physical Activity for Health Research Centre, Health Research Institute, Department of Physical Education and Sport Sciences University of Limerick Limerick Ireland; ^3^ Leibniz Institute for Prevention Research and Epidemiology – BIPS Bremen Germany; ^4^ Institute of Sport Sciences Université de Lausanne Lausanne Switzerland; ^5^ Senate Department for Science, Health and Care Berlin Germany

**Keywords:** childcare, health promotion, physical activity, public policy, systematic review

## Abstract

**Background:**

Early childhood education and care (ECEC) is crucial for shaping physical activity (PA) behaviours due to the significant time children spend in this setting. In addition, research has shown that public policies can be an important means to create a healthy environment. This systematic review explores the effectiveness of public policies promoting PA in ECEC.

**Methods:**

Nine online databases (Scopus, Pubmed, Web of Science, SportDiscus, Cinahl, IBSS, ERIC, APA PsychINFO and Cochrane library) were searched in August 2023 for studies that examined public policies impacting children's PA behaviour or environments in ECEC. Data were extracted, and a quality assessment was performed using the Downs and Black checklist, and a narrative synthesis was applied.

**Results:**

A total of 11 articles met the inclusion criteria. The studies from the United States, Canada and Australia involved either the implementation of legally binding policies (*n* = 6) or voluntary accreditation standards (*n* = 5). Studies reported on the adherence of ECEC centres to policies, the effects on children's PA behaviour or changes in the environment using both device‐based (e.g., accelerometer) and self‐report (e.g., questionnaires and interviews) measures as well as observation. Reported adherence rates of childcare centres to new regulations ranged from 74% to 94%. Studies on policies that implemented PA into the curriculum required a mandatory PA time of at least 60 min per day or implemented revised accreditation standards reported positive effects on the ECEC centres PA environment. Effects on the PA behaviour of children remained inconclusive, with studies reporting on both increased and decreased PA levels.

**Conclusion:**

Public policies have the potential to change the environment and positively influence PA behaviour in preschool children. However, due to the heterogenous methodological approaches in the identified studies, the findings of this review have certain limitations. Future research needs to further investigate the effectiveness of policy approaches to promote PA in early childhood settings.

**Summary**

- Public policies have the potential to change the environment and positively influence physical activity behaviours in preschool children.
- Positive effects were identified for public policies that included physical activity in the curriculum, required a minimum of 60 min of physical activity per day or revised accreditation standards.
- However, these results must be interpreted with caution, as the heterogeneity of methodological approaches poses a challenge for assessing the effectiveness of policies on the physical activity (PA) behaviour of children in the early childhood education and care setting.
- A better understanding of the causal relationships between public policies and behaviour would not only benefit research but could also inform evidence‐based policy making.

## 1 Introduction

Early childhood is recognised as an important period that influences wellbeing throughout the life course (Rossin‐Slater 2015). According to the World Health Organization (WHO), early childhood is a time in which children rapidly develop cognitive, physical, language, motor, social and emotional competences (WHO 2019, 2020).

WHO guidelines on physical activity (PA) for children under 5 years of age recommend engaging in a minimum of 180 min of PA daily (starting at the age of one), of which, starting from the age of 3, at least 60 min should be of moderate to vigorous intensity (WHO 2019). Additionally, children aged 3 or 4 should accumulate less than 1 h of recreational screen time per day and get 10–13 h of good quality sleep (WHO 2019). Scientific evidence has demonstrated the positive effects of achieving these guidelines on weight status, bone health, cardiometabolic health and cognition in children under the age of 6 (Carson et al. 2017; Pate et al. 2019).

However, a systematic review and meta‐analysis by Tapia‐Serrano et al. (2022) based on data from 11 768 preschoolers (3–5 years old) indicated that only 11.3% of children adhered to these guidelines on PA, sedentary behaviour and sleep. Specific data on the PA behaviour of children aged 3 to 6 from Germany showed that 43% of girls and 49% of boys met WHO guidelines of 60 min of moderate‐to‐vigorous physical activity (MVPA) per day (Robert Koch‐Institut 2020).

According to several studies, early childhood education and care (ECEC) centres such as kindergartens, nursery schools or preschools play a prominent role in influencing the PA behaviour of preschool children (Bower et al. 2008; Goldfield et al. 2012). In the United States, study data showed that most infants (72%) receive nonparental childcare within the first year of life (Huston, Bobbitt, and Bentley 2015). In the European Union, approximately 89% of children from the age of 3 attend formal childcare (Eurostat 2024). This number is even higher in some countries such as Germany, where 92% of children from the age of 3 attend kindergarten until entering primary school (Bundesministerium für Familie, Senioren, Frauen and Jugend 2023). Considering the fact that children spend between 25–40 h per week in ECEC (Bundesministerium für Familie, Senioren, Frauen and Jugend 2023; Story, Kaphingst, and French 2006), this setting can have a major influence on their health through education and PA promotion measures (e.g., active play time, PA‐promoting environments and staff attitude towards PA).

A substantial body of literature on PA promotion in ECEC exists. Several studies reported on the significant improvement of motor skills in healthy preschool children due to PA promoting interventions in this setting (Gordon et al. 2013; Li, Liu, and Ying 2022). According to the meta‐analysis provided by Gordon et al. (2013), teacher‐led PA offers have proven to be more successful in increasing preschoolers' MVPA than offers led by parents. Further, they reported that outdoor interventions and structural changes in ECEC had the largest effect on children's MVPA. A more recent study by Tonge, Jones, and Okely (2020) also showed that children in ECEC centres that offered free routines, that is, that allowed children to move freely between indoor and outdoor environments, spent more time in total PA and in MVPA. In addition, a review by Trost, Ward, and Senso (2010) indicated that the education and training of ECEC centres' staff and their behaviour on‐site significantly influence the MVPA of children.

However, ECEC centres often face challenges implementing PA interventions due to limited or missing PA‐friendly spaces (e.g., gymnasiums and swimming pools), limited financial resources, limited staff qualifications or excessively rigid daily routines that often result in the cancellation of physical education (Wolbring et al. 2021). Therefore, to sustainably change the PA behaviour of preschool children, it has been suggested to change ECEC centres' curricula (Goldfield et al. 2012; Li, Liu, and Ying 2022). This is in line with policy documents that highlight the need for PA promotion in ECEC. In particular, WHO's Global Action Plan for PA (WHO 2018) and the PA strategy for the WHO European Region (WHO 2016) recommend establishing PA‐friendly environments in ECEC, developing guidelines and programs for PA promotion and including PA promotion in teacher education.

In recent years, the importance of public policies to promote PA has been widely acknowledged, as they can “change systems instead of individuals”, create supporting contexts and support individuals to adopt and maintain health behaviours (Lakerveld et al. 2020). Numerous studies have analysed PA policies (Klepac Pogrmilovic et al. 2018) and shown their effectiveness (Gelius et al. 2020). While there is growing evidence for the effect of PA promoting policies in several areas, including the school setting (Woods et al. 2021), transportation (Zukowska et al. 2022), mass media campaigns (den Braver et al. 2022) and sport participation (Volf et al. 2022), no systematic review has been conducted on the effectiveness of PA promoting policies in ECEC.

Therefore, this study aims to synthesise the scientific literature on the effects of PA promoting public policies in the ECEC setting. In this context, we define public policy as any “decisions, plans, and actions that are enforced by national or regional governments or their agencies (including at the local level) which may directly or indirectly achieve specific health goals within a society” (Lakerveld et al. 2020). It is investigated whether these policies influence the PA behaviour of preschool aged children (0–6 years) or help change the PA environment in ECEC. Specifically, this review will answer the following research question: Which impact was achieved by public policies on preschool‐children's PA behaviour or the PA promoting environment (incl. Adherence to public policies) in the ECEC setting?

## 2 Methods

To answer the research question, this systematic review adapted the Policy Evaluation Network (PEN) protocol for systematic literature reviews (Volf et al. 2020). The review follows the Preferred Reporting Items for Systematic Reviews and Meta‐Analysis (PRISMA) statement (Page et al. 2021).

### 2.1 Search Strategy

A systematic search was performed in nine databases in August 2023: Scopus, Pubmed, Web of Science, SportDiscus, Cinahl, IBSS, ERIC, APA PsychINFO and the Cochrane library. Additionally, the reference lists of included articles were screened manually to identify relevant publications.

As displayed in Table 1, a search term was created in collaboration with a librarian. It combined the three keywords: (1) policy, (2) physical activity and (3) childcare, as well as their synonyms and closely related words.

**TABLE 1:** Search terms.

Keyword	Synonyms
Policy	OR (policies) OR (framework*) OR (legislation*) OR (curricul*)
Physical activit*	OR (physical inactivity) OR (“physical education”) OR (sedentary) OR (sitting) OR (exercise)
Childcare*	OR (“child care”) OR (child‐care*) OR (preschool*) OR (pre‐school*) OR (nurser*) OR (playschool*) OR (kindergarten*) or (daycare*) OR (“day care*”) OR (day‐care*) OR (“early childhood”)

### 2.2 Eligibility Criteria

1. Studies were included in this review if they fulfilled the following eligibility criteria: The study analysed public policies.
2. The study analysed PA behaviour (e.g., device‐based or self‐report assessment of PA levels) or changes in features of the physical and social PA environment (e.g., change in availability of PA promoting equipment or staff behaviour) as the outcome.
3. The study focused on the setting of ECEC.
4. The study was published in a peer‐reviewed scientific journal.

No limits were set to study design, publication date or language.

### 2.3 Study Selection

In a first step, two authors screened studies independently for eligibility based on their title‐abstract; discrepancies were discussed until consensus was reached. Subsequently, two researchers independently screened full texts for eligibility. Differences were discussed with a third author until consensus was reached.

To facilitate the screening process, the systematic review software Covidence was used (Veritas Health Innovation, Melbourne, Australia; www.covidence.org).

### 2.4 Data Extraction

Data were extracted by one member of the researcher team, whose work was subsequently checked by a second member. A Microsoft Excel spreadsheet was used to store extracted data. Extraction included information on (1) the study population (i.e., age group, sample size and study location), (2) the policy intervention (i.e., the policy action, sectors involved in preparation and implementation, legal status, initial aim, timeframe and allocated budget), (3) the study type (incl. Type of measurement) and (4) the outcome of the intervention (effect on the environment, effect on PA behaviour).

### 2.5 Quality Assessment and Data Analysis

The quality of the included studies was assessed independently by two researchers using an adapted version of the Downs and Black checklist (Downs and Black 1998) (Supporting Information). Discrepancies were discussed until reaching consensus.

A narrative synthesis of the studies was performed to interpret and analyse the data. Core characteristics of each article were systematically described, including the population (age, number, additional information), intervention (policy action, sectors/institutions involved in preparation, sectors/institutions involved in implementation, implementation plan, legal status, target groups, goals and targets, timeframe, budget, evaluation and surveillance), comparison (control group), outcome (measurement type, measurement instrument, effect on behaviour, effect on environment) and other aspects (study design, duration).

## 3 Results

### 3.1 Study Selection

**FIGURE 1:**
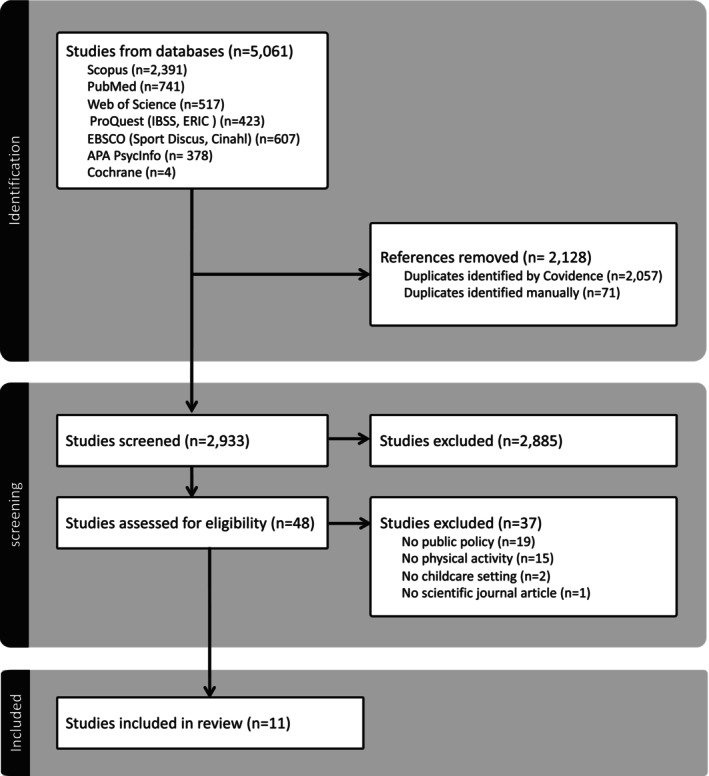
PRISMA flowchart of study inclusion.

The search identified a total of 5061 articles, 2128 of which were removed as duplicates. In a first step, titles and abstracts of 2933 articles were screened, of which 48 were deemed to be eligible for full text review. A total of 37 articles were excluded: 19 did not analyse a public policy, 15 did not measure PA outcomes, two did not focus on ECEC and one did not match the criterion of being published in a peer‐reviewed scientific journal. Eleven articles met the inclusion criteria. A flowchart displaying the search and screening sequence is displayed in Figure 1.

### 3.2 Study Sample Characteristics

As presented in Table 2, studies included in this review were mainly performed in the United States of America (64%) (Benjamin Neelon et al. 2017; Chang‐Martinez et al. 2018; Kakietek et al. 2014; Kracht, Webster, and Staiano 2020; Lyn et al. 2013; O'Neill et al. 2017; Stephens et al. 2014). Approximately 27% of studies were implemented in Canada (Carson et al. 2015; Carson et al. 2022; Tugault‐Lafleur et al. 2022) and 10% in Australia (Yoong et al. 2016).

**TABLE 2:** Study characteristics.

References	Country	Policy level/legal status	Study design	Study population				CG	Quality rating
				Children	Age group	Staff	ECEC Centres		
Benjamin Neelon et al. (2017)	USA	State/binding	Longitudinal	T1–6 IG:180 T1–6 CG: 180	3–5	—	T1–6 IG: 20 T1–6 CG: 20	🗸	72%
Carson et al. (2015)	Canada	State/nonbinding	Pre–post	T1/T2: 141	1,5–5	—	T1/2:8		72%
Carson et al. (2022)	Canada	State/nonbinding	Pre–post	T1/T2 IG: 126 T1/T2 CG: 126	1,5–5	72	IG: 11 CG: 8	🗸	78%
Chang‐Martinez et al. (2018)	USA	State/binding	Cross‐sectional	—	2–5	—	34		56%
Kakietek et al. (2014)	USA	City/binding	Cross‐sectional	—	3–4	—	174		67%
Kracht, Webster, and Staiano (2020)	USA	State/binding	Pre–post	T1: 112 T2: 83	3–4	T1: 22(6) T2: 22(7) per ECEC centre^a^	T1/T2: 9		78%
Lyn et al. (2013)	USA	State/nonbinding^b^	Pre–post	2042^c^	2–5	—	T1/T2: 22		67%
O'Neill et al. (2017)	USA	State/nonbinding	Pre–post	—	3–5	—	IG: 33 CG: 26	🗸	61%
Stephens et al. (2014)	USA	City/binding	Cross‐sectional	1352	2–5	—	110		78%
Tugault‐Lafleur et al. (2022)	Canada	State/binding	Pre–post	—	2,5–5	T1: 910 T2: 549	T1: 592 T2: 378		61%
Yoong et al. (2016)	Australia	National/nonbinding	Longitudinal	T1: not assessed T2: 68 (43) T3: 70 (70) T4: 71 (35) per ECEC centre^a^	0–5	—	Total: 358 T1: 279 T2: 238 T3: 255 T4: 227		44%

Abbreviations: CG, control group; ECEC Early Childhood Education and Care; IG, intervention group.

^a^ Only reported as mean (SD).

^b^ Regulations were mandatory for schools.

^c^ Children visited ECEC centres but were not investigated.

The studies encompassed a total sample of approximately 1780 ECEC centres, 4502 children and 1729 educators. The scope of the individual studies varied, involving between eight (Carson et al. 2015) and 970 centres (Tugault‐Lafleur et al. 2022), 141 (Carson et al. 2015) to 2042 children (Lyn et al. 2013) and 72 (Carson et al. 2022) to 1459 educators (Tugault‐Lafleur et al. 2022). Notably, two of the studies also incorporated parental perspectives in their data collection (Carson et al. 2015; Carson et al. 2022). The age of the children studied ranged from 0 to 5 years, with most studies (*n* = 8) commencing at ages 2 to 3 years. Only one study included the full age range of 0 to 5 years (Yoong et al. 2016), while Kakietek et al. (2014) and Kracht, Webster, and Staiano (2020) focused on the narrower age range of 3 to 4 years.

### 3.3 Study Design and Quality

Among the eleven studies, six were pre–post studies (Carson et al. 2015; Carson et al. 2022; Kracht, Webster, and Staiano 2020; Lyn et al. 2013; O'Neill et al. 2017; Tugault‐Lafleur et al. 2022), three were cross‐sectional studies (Chang‐Martinez et al. 2018; Kakietek et al. 2014; Stephens et al. 2014) and two implemented a longitudinal evaluation (Benjamin Neelon et al. 2017; Yoong et al. 2016). Three studies included a control group (Benjamin Neelon et al. 2017; Carson et al. 2022; O'Neill et al. 2017). ECEC centres used as control groups were chosen based on their location in areas with similar geographic location, urban density, and climate.

The quality assessment (Table 2) of the studies indicates the entire range from poor (*n* = 2) to fair (*n* = 6) and good quality (*n* = 3) according to the adapted Downs and Black (1998) quality assessment tool.

### 3.4 Type of Policy

The policy actions examined in the studies focused on either new regulations or revisions of existing standards, such as accreditation standards or state licensing standards. Most policies were implemented at state level (*n* = 8), while two studies focused on city level and one on the implementation of new accreditation standards at national level. Among the included studies, six policies were legally binding for all ECEC centres in the addressed area, while five were part of nonbinding accreditation standards. As shown in Table 1, the studies investigated a variety of policy contents. These included the implementation of mandatory daily PA time (*n* = 6) (Benjamin Neelon et al. 2017; Kakietek et al. 2014; Kracht, Webster, and Staiano 2020; Lyn et al. 2013; Stephens et al. 2014; Tugault‐Lafleur et al. 2022), daily structured PA time (*n* = 3) (Kakietek et al. 2014; O'Neill et al. 2017; Stephens et al. 2014), the inclusion of PA in the ECEC curriculum (*n* = 6) (Carson et al. 2015; Carson et al. 2022; Chang‐Martinez et al. 2018; Lyn et al. 2013; O'Neill et al. 2017; Yoong et al. 2016) or the participation in mandatory training on PA in ECEC (*n* = 2) (O'Neill et al. 2017; Yoong et al. 2016). In two instances, new regulations also stipulated that PA should not be used as a form of punishment or reward in ECEC (Lyn et al. 2013; O'Neill et al. 2017). Binding policies primarily focused on mandatory PA time and specified the required number of PA minutes per day. In contrast, voluntary policies were more detailed, specifying the actions and regulations that should be integrated into the daily routines of ECEC centres to qualify for accreditation.

### 3.5 Type of Measurement

The studies included in this review employed a variety of methods to assess either the PA behaviour or behaviour change among children (Benjamin Neelon et al. 2017; Carson et al. 2015; Carson et al. 2022), environmental status quo or changes (Kakietek et al. 2014; Lyn et al. 2013; O'Neill et al. 2017; Tugault‐Lafleur et al. 2022; Yoong et al. 2016) or both (Chang‐Martinez et al. 2018; Kracht, Webster, and Staiano 2020; Stephens et al. 2014). These measurements were used to analyse compliance with or the effects of new regulations or revised accreditation standards.

Device‐based, self‐report or observational measurement methods were used in the studies. Four studies used more than one measurement method: Three of these studies used device‐based, self‐report and observational measures to assess both PA behaviour and the PA environment (Carson et al. 2022; Kracht, Webster, and Staiano 2020; Stephens et al. 2014), while one study used self‐report and observation measures to assess the PA environment (Kakietek et al. 2014). Studies that were based on one measurement method include a device‐based study that assessed PA behaviour (Carson et al. 2015), two studies that used self‐report measures to assess the PA environment (Tugault‐Lafleur et al. 2022; Yoong et al. 2016) and four studies that used observational measures to assess either the PA behaviour (Benjamin Neelon et al. 2017) or the PA environment (Chang‐Martinez et al. 2018; Lyn et al. 2013; O'Neill et al. 2017).

As displayed in Table 3, all four studies that used device‐based measures are based on data collected through accelerometers (Carson et al. 2015; Carson et al. 2022; Kracht, Webster, and Staiano 2020; Stephens et al. 2014).

**TABLE 3:** Implemented policies, measurements and outcomes.

References	Policy	Policy content	Measurement	Effect on PA behaviour	Effect on PA environment
Benjamin Neelon et al. (2017)	**New regulation**	Mandatory PA time (at least 60 min of PA/day)	*Observation:* System for recording activity in preschoolers	*Preschoolers (3–5 years old):* ↑ light PA in IG and CG ↓ moderate PA in IG o vigorous PA in IG and CG ↓ total MVPA in IG ↑ sedentary time	n.a.
Carson et al. (2015)	Revision of accreditation standards	Integration of PA promotion into the childcare curriculum: Promotion of physical literacy and reduction of sedentary behaviour	*Device‐based:* Accelerometer (Philips Respironics)	*Toddlers (1,5 < 3‐ year old):* ↓ sedentary time o light PA ↑ total MVPA *Preschoolers (3–5 years old):* ↑ sedentary time ↓ light PA o total MVPA	n.a.
Carson et al. (2022)	Revision of accreditation standards	Integration of PA promotion into the childcare curriculum: Promotion of physical literacy and reduction of sedentary behaviour	*Device‐based:* Accelerometer (ActiGraph wGT3X‐BT) *Self‐report:* HATCH educator questionnaire HATCH parents' questionnaire *Observation:* EPAO (PA components only), Movement Environment Rating Scale MOVERS, Children's Physical Environments Rating Scale CPERS5	*Toddlers and preschoolers (1,5–5 years old):* o sedentary time o LPA o MVPA	⇡ EPAO PA Score (no differences between IG & CG) ⇡ MOVERS score (no differences between IG & CG)
Chang‐Martinez et al. (2018)	**New regulations** ^a^	Integration of active play into the childcare curriculum	*Observation:* EPAO (selected components only) Number of occasions and duration in minutes of total active play time	n.a.	94.1% adherence to PA regulations indoor and outdoor
Kakietek et al. (2014)	**New regulations** ^a^	Mandatory PA time (at least 60 min of total PA/day) Daily structured PA time (at least 30 min of structured PA/day)	*Self‐report:* in‐person interviews *Observation:* site inventory (availability/access to play spaces)	n.a.	85.5% centres provide at least 60 min of PA/day 77.5% centres provide at least 30 min of structured PA/day 74% of centres are compliant to PA regulations
Kracht, Webster, and Staiano (2020)	**Revision of state licensing requirements**	Mandatory PA time (at least 60 min of PA/day)	*Device‐based:* Accelerometer (ActiGraph GT3X+) *Self‐report:* baseline questionnaire on policy engagement *Observation:* EPAO	*Preschoolers (3–4 years old):* ↑ sedentary time ↓ total PA o total MVPA higher active play → lower SB and higher total PA	o total EPAO score ⇡ active play time ↑ active opportunities ⇣ sedentary opportunities ⇣ sedentary environment ⇡ portable play environment ⇣ fixed play environment ↑ staff behaviour ⇣ PA training and education o PA Policy ↑ active opportunities and staff behaviour score
Lyn et al. (2013)	New regulations^a^, ^b^	Integration of PA promotion into the childcare curriculum Mandatory PA time (at least 60 min of PA/day) Regulation not to use PA as punishment or reward Requirement to meet the National Association for Sport and Physical Education guidelines	*Observation:* EPAO	n.a.	↑ overall PA score ↑ active play ↑ portable play environment ↑ staff behaviour ↑ PA training and education ↑ sedentary environment o sedentary behaviour o fixed environment
O'Neill et al. (2017)	Revision of accreditation standards	Aim to encourage children to be active indoors and outdoors Adoption of a written PA policy Participation in PA training at least once per year for ECEC staff Regulation not to withhold PA as punishment. Daily structured PA time (5–10 min of structured PA 2 or more times/day) Mandatory PA time (active outdoor play for 90–120 min/day). Provision of a variety of play materials to promote PA indoors. Provision of a variety of play materials to promote PA	*Observation:* EPAO	n.a.	⇡ overall EPAO PA‐score with no significant differences between groups ⇣ active opportunities (IG&CG) ⇡ sedentary opportunities (IG&CG) ⇡ staff behaviour (IG&CG) ⇡ PA policy sedentary environment: ⇡ IG ⇣ CG portable play environment: ⇡ IG ⇣ CG fixed environment: ↑ IG ↓CG PA training and education: ⇡ IG ↓CG
Stephens et al. (2014)	**New regulations** ^a^	Mandatory PA time (at least 60 min of total PA/day Daily structured PA time (at least 30 min of structured PA/day)	*Device‐based:* Accelerometer (ActiGraph GT3X) *Self‐report:* staff report *Observation:* staff and PA offers	*Preschoolers (2.8–5.9 years old)* boys → more MVPA than girls compliance to regulations reported in subjective and objective measurement → more time in MVPA time in MVPA ↛ compliance with 30 min of structured PA/day time in MVPA → with compliance with 60 min of total PA/day	78.5% centres provide at least 30 min of structured PA/day (subjective measurement) 29.9% centres provide at least 30 min of structured PA/day (objective measurement) 87.2% centres provide at least 60 min of total PA/day (subjective measurement) 25.7% centres provide at least 60 min of total PA/day (objective measurement)
Tugault‐Lafleur et al. (2022)	**New regulations** ^a^	Mandatory PA time (at least 60 min of outdoor play/day) Movement skills and injury prevention in all PA activities	*Self‐report:* adapted EPAO‐Self‐Report incl. Report on PA practices	n.a.	*Policies:* ↑ provision of activities ↑ total amount of active play ↑ staff‐led activities ↑ free play ↑ outdoor play time ↑ staff Training on PA and physical literacy ↑ breaking up prolonged sitting *Practices:* o daily activities to develop movement skills ↑ at least 120 min of PA/day ↑ at least 60 min outdoor play/day ↑ less than 30 min screen time o staff being active o no prolonged sitting periods o learn why PA is good
Yoong et al. (2016)	Revision of accreditation standards^a^	Integration of PA promotion into the childcare curriculum Provision of a scheduled time for PA/day Provision of a scheduled time for outdoor PA Participation in PA training at least once per year for ECEC staff	*Self‐report:* telephone survey	n.a.	↑ written PA policy ↑ daily movement skill sessions ↑ trained staff past year o outdoor active play for ≥25%

*Note:* Bold indicates legally binding policy.

Abbreviations: CG control group; EPAO, Environment and Policy Assessment and Observation; HATCH, Healthy physical AcTive Childcare; IG, intervention group; MVPA, moderate to vigorous PA; n.a., not applicable; PA, physical activity; MOVERS, Movement Environment Rating Scale ↑ significant improvement; ⇡improvement without significance; ↓ significant decrease; ⇣ decrease without significance; o no effect/change; → associated with; ↛ not associated with.

^a^ Additionally implemented regulations for nutrition.

^b^ Regulations were mandatory for schools.

Out of the six studies that used self‐report measures, three studies used surveys (Carson et al. 2022; Kracht, Webster, and Staiano 2020; Tugault‐Lafleur et al. 2022), two interviews (Kakietek et al. 2014; Yoong et al. 2016) and one staff reports (Stephens et al. 2014).

Five of the eight studies that used observational measures applied the Environment and Policy Assessment and Observation (EPAO) tool (Carson et al. 2022; Chang‐Martinez et al. 2018; Kracht, Webster, and Staiano 2020; Lyn et al. 2013; O'Neill et al. 2017). The EPAO tool combines a total of 16 subscales, eight of which focus on PA‐related areas that are combined into a total EPAO PA‐Score (Ward 2008). In addition, studies utilised the Observation System for Recording Activity in Preschoolers developed by Brown et al. 2006 (Benjamin Neelon et al. 2017), the Movement Environment Rating Scale MOVERS developed by Archer and Siraj 2017 (Carson et al. 2022), the Children's Physical Environments Rating Scale CPERS5 developed by Moore and Sugiyama 2007 (Carson et al. 2022) and/or additional observational measures developed for the respective study (Chang‐Martinez et al. 2018; Kakietek et al. 2014; Stephens et al. 2014).

### 3.6 Effects on PA Behaviour

The studies paint an ambiguous picture of the effects of new regulations, changes to existing regulations or updates of accreditation standards on the actual PA behaviour of children in ECEC.

Three studies provided evidence on the effectiveness of new regulations mandating at least 60 min of PA per day, reporting inconclusive results. Two of these studies used device‐based measures to quantify effects on PA behaviour (Kracht, Webster, and Staiano 2020; Stephens et al. 2014), and one was based on observation (Benjamin Neelon et al. 2017). The observational study showed that a mandatory PA time of at least 60 min per day resulted in a decrease in moderate PA and total MVPA. Both the intervention and control groups in this study showed an increase in light PA and an increase in sedentary time (Benjamin Neelon et al. 2017). Similar findings were reported in the device‐based study by Kracht, Webster, and Staiano (2020): While there was an overall increase in sedentary time, the level of MVPA remained constant. Nonetheless, this study identified a positive association between more time allocated for active play and the duration of children being moderately or vigorously active. The device‐based study conducted by Stephens et al. (2014) reported that there was a statistically significant association between ECEC centres' compliance with the requirement of 60 min of total PA per day and the amount of time children spent in MVPA. There was, however, no detectable association between meeting the requirement of 30 min of structured PA and time spent in MVPA. The study also reported that boys spent significantly more time in MVPA than girls but not whether the policy had any effect on these gender differences (Stephens et al. 2014).

Two device‐based studies analysed the effects of revised nonbinding accreditation standards on children's PA behaviour in eight licensed child care centres in Alberta. The study by Carson et al. (2015) showed an increase in total MVPA and a reduction in sedentary behaviour among toddlers, while preschoolers increased sedentary behaviour, decreased light PA (LPA) and had no changes in MVPA. A follow‐up study, however, did not identify any significant changes in LPA, MVPA and sedentary time for both age groups (Carson et al. 2022).

### 3.7 Effects on the PA Environment

The analysis of effects on the environment of ECEC centres indicated an overall adherence to new regulations. At the state level, new binding regulations resulted in a 91% adherence rate to new curricula and the implementation of daily active play in ECEC centres (Chang‐Martinez et al. 2018). The study by Kakietek et al. (2014) indicated an 85.5% adherence rate to the requirement of 60 min of PA per day and a 77.5% adherence rate for providing 30 min of structured PA per day. The inclusion of a mandatory minimum of 60 min of active play in ECEC centres significantly increased active opportunities for children and positively influenced staff behaviour (Kracht, Webster, and Staiano 2020). However, the study by Stephens et al. (2014) highlighted a significant discrepancy in the measurement of regulation adherence. While 78.5% of ECEC centres self‐reported providing at least 30 min of structured PA, observational (objective) measurements indicated that only 29.9% implemented this regulation. An even greater discrepancy was observed for the requirement of at least 60 min of total PA per day, showing a 61.5%‐points difference between self‐report (subjective) and observational (objective) measurement.

Adherence to binding state‐level regulations that mandate at least 60 min of outdoor play, the promotion of movement skills and measures for injury prevention significantly increased ECEC centres action regarding total active playtime, staff‐led activities, free play, outdoor playtime, staff training and breaking up prolonged sitting periods (Tugault‐Lafleur et al. 2022). Additionally, there was a significant improvement in practices ensuring at least 120 min of PA per day and at least 60 min of outdoor play.

The implementation of nonbinding accreditation standards was reported to have positive effects on the environment. Lyn et al. (2013) detected a significant increase in the overall EPAO PA‐score, the duration and frequency of active play, portable play environment (e.g., balls and riding toys), staff behaviour (e.g., staff joining or encouraging PA), staff PA training and education and nonsedentary environments (e.g., no TV or computer available in the play room and PA‐promoting prompts like posters or books).

The controlled study by O'Neill et al. (2017) compared the effects of PA standards in South Carolina (which adopted new mandatory PA standards as part of its child care quality enhancement program) and North Carolina (which did not adopt a new policy). The results show that revised accreditation standards led to an overall increase in the EPAO‐based PA‐score in both the intervention and control groups. Both groups experienced an increase in ECEC PA policies, staff behaviour and sedentary opportunities. Changes, though not statistically significant, were also observed in the sedentary and portable play environments, with the intervention group showing an increase and the control group showing a decrease in portable play environment. In addition, the intervention group showed a decrease, and the control group showed an increase in the sedentary environment, indicating positive effects on the PA‐friendly environment of the intervention group. Significant positive changes were only observed in the score for the *fixed environment* item.

Significantly positive effects were also reported in the longitudinal telephone survey by Yoong et al. (2016), which investigated the environmental change triggered by a revision of accreditation standards. These changes included improvements in written PA policy, the implementation of daily PA sessions and the training of PA staff within the past year. The study identified no significant difference in the proportion of services implementing each policy by locality or socio‐economic characteristics of the childcare service.

### 3.8 Environment and Behaviour

Three studies analysed the effects of public policies on the PA environment in ECEC and its subsequent impact on children's PA behaviour, showing inconclusive results. Carson et al. (2022) investigated the influence of environmental changes related to revised accreditation standards (based on MOVERS and EPAO Scores) on the PA behaviour, finding no statistically significant influence. According to the study by Stephens et al. (2014), the time children spent in MVPA was associated with the availability of dedicated outdoor play spaces in a statistically significant way. Children attending ECEC with a dedicated PA space showed an increase of MVPA by nearly 1 min per hour. Kracht, Webster, and Staiano (2020) identified environmental changes in ECEC after the revision of state licensing requirements. Of these changes, especially, the increase of active play minutes was able to significantly increase children's total PA.

## 4 Discussion

### 4.1 Main Findings

This review presents evidence on public policies for the promotion of PA in the ECEC setting and the effects of these policies on both preschool children's PA behaviour and the PA environment of ECEC centres.

Different types of policies were identified, such as the implementation of new binding regulations for ECEC centres (e.g., mandatory daily/structured PA time) and the revision of nonbinding accreditation standards (e.g, incorporation of PA promotion into the ECEC curriculum, training of ECEC staff on implementing PA promotion). The included studies measured the effects of public policies on various outcome variables, including the PA behaviour of children, the PA environment or ECEC centres' adherence to the policies.

Several studies reported high adherence rates of ECEC centres to binding PA policies of 74–94%. Positive effects of revised accreditation standards on the PA environment (e.g., increase in portable play environment, decrease of sedentary environment, improvements in staff behaviour) were identified using the EPAO tool. However, negative effects on fixed play environment as well as on PA training and education were also identified. The impact of policies on children's PA behaviour remained inconclusive. While one study reported an increase in MVPA for toddlers aged 19–35 months, other studies reported no change of MVPA or even a decrease in MVPA or total PA. Positive effects on time spent in MVPA were identified for policies that allocated more time to active play, thus contributing to the achievement of the WHO guidelines of 60 min of MVPA per day.

Due to methodological issues and the fair to poor quality of most studies, these results should be treated with caution. Regarding the adherence rate to PA policies, one study reported a great discrepancy between objective measurement (observation) and subjective reports (by ECEC staff). In addition, studies with a control group showed that changes in PA behaviour cannot be solely attributed to policy actions.

### 4.2 Comparison to Other Studies

The results need to be interpreted in the light of previous studies on PA in ECEC. For instance, some of the included studies identified positive effects (particularly on the PA environment) of policies which integrated PA into the curriculum. The importance of incorporating PA into preschool curricula has been previously highlighted in numerous studies (Goldfield et al. 2012; Kreichauf et al. 2012; Li, Liu, and Ying 2022; Loy‐Ee and Ng 2018; Trost, Fees, and Dzewaltowski 2008). An important aspect in this context is that PA “should be integrated into the daily routines and the existing curriculum of preschools and [should] not be seen as something that is competing with other educational goals” (Kreichauf et al. 2012).

This review also showed that policies that include a mandatory PA time of at least 60 min per day have positive effects on the PA environment in ECEC. In this context, it should be noted that children engage in MVPA for only about 27% of their playtime (Dowda et al. 2004). As a consequence, Dowda et al. (2004) have recommended that PA should be incorporated into the daily preschool schedule through multiple shorter activity sessions of 15–20 min to achieve the recommended 60 min of MVPA per day (Dowda et al. 2004). Another review has shown that MVPA levels of children doubled when children played outdoors versus indoors; in addition, outdoor play space and outdoor portable equipment were associated with increased MVPA (Martin et al. 2022). This suggest that policies stipulating a mandatory PA time of at least 60 min per day should recommend explicitly to implement this requirement through several shorter sessions during the day, through outdoor play time and supported through outdoor portable equipment.

Results additionally indicate that the change of policies had a positive influence on staff behaviour and the training of staff in the field of PA promotion (O'Neill et al. 2017; Lyn et al. 2013; Kracht, Webster, and Staiano 2020). While the included studies did not analyse the effect of staff behaviour on children's PA behaviour, other studies focused specifically on this aspect: A device‐based study has shown a significant association between ECEC centres staff sedentary behaviour on‐site and children's sedentary behaviour but not significant assocations for PA (Tonge, Jones, and Okely 2021). Another device‐based study showed that children spent significantly more time in MVPA in ECEC centres where the staff had a high intention in relation to PA learning experiences compared to centres with lower intentionality (Veldman et al. 2018).

Furthermore, our results indicate that accreditation standards can lead to an increase in portable play environment (e.g., balls, riding toys). Importantly, other studies have shown that portable play environment can significantly increase children's PA behaviour (Coe 2020; Terrón‐Pérez et al. 2021), suggesting that such policies might be effective. Another study by Chen et al. (2020) has shown that preschools' structural prerequisites, such as their own organisational PA policies, can increase children's PA levels. In other words, it has been shown in the scientific literature that policies and changes of the PA environment in ECEC can have a positive effect on the PA behaviour of young children.

### 4.3 Recommendations for Research and Practice

From a methodological perspective, it is worth noting that significant discrepancies in measuring regulation adherence were identified (Stephens et al. 2014). While subjective reports indicate that a large majority of ECEC centres provide at least 30 min of structured PA per day or 60 min of total PA per day (78.5%/87.2%), objective measurements show that these regulations are only fulfilled by a minority of ECEC centres (29.9%/25.7%). This has important implications for policy and practice, as it may indicate that ECEC staff have difficulties assessing the time children are active. Consequently, problems related to the subjective assessment of children's PA may be a barrier to policy implementation in ECEC centres. This limitation needs to be addressed by policymakers (e.g., by providing guidance through implementation toolkits) and researchers (e.g., by measuring policy implementation objectively).

This review also shows that the literature on PA‐promoting ECEC policies remains limited; however, lessons can be drawn from similar research in other domains. For example, a recent review on the impact of PA policies within the school setting identified evidence on the effectiveness of policy in nine areas: whole‐of‐school approaches, physical education, sport/extracurricular PA, classroom‐based PA, active breaks/recess, physical environment, shared‐use agreements, active school transport and surveillance (Woods et al. 2021). Although this review refers to a setting with different aims and older children, some of the findings may be transferable. In particular, ECEC policies on mandatory daily PA time/structured PA time might be comparable to school policies related to physical education, sport and extracurricular PA and classroom‐based PA. However, policies on active transport to school may not be transferable, as (active) transport to ECEC for younger children may be more dependent on the parents. Nevertheless, future research on the effectiveness of PA policies in the ECEC setting could explore whether results from the comparatively well‐researched school setting are transferable.

To translate research into practice, as suggested by Sallis and colleagues in the Behavioural Epidemiology Framework (Sallis, Owen, and Fotheringham 2000), more research on the effectiveness of policy interventions in the ECEC setting would be beneficial. While some studies have found positive effects of policies on the PA environment, it is still difficult to draw general conclusions due to the limited number and heterogeneous methodology of the included studies. In particular, there is limited evidence on gender‐specific and equity‐related effects of public policies. Furthermore, there is a lack of studies from other countries besides the United States, Canada and Australia. A stronger evidence base could also help to formulate more specific recommendations for promoting PA in the ECEC setting. Up to now, most policy documents include a very limited number of recommendations on PA promotion in ECEC (such as WHO's Global Action Plan on PA; WHO 2018) or combine them with recommendations for the school setting (such as the PA Strategy for the WHO European Region 2016–2025; WHO 2016). Consequently, robust evidence could help to increase the recognition of ECEC as a distinct setting for PA promotion, with different needs from the school setting.

### 4.4 Limitations

This review has several limitations. Importantly, the empirical findings are based on studies that were conducted in three countries. As a consequence, the results might not be fully applicable to other geographical locations or cultural contexts. In addition, the comparability of the included studies is limited as they applied different methodological approaches. It is, however, a strength of this review that it covers public policies that are binding and nonbinding, implemented on different levels (city, state or country level) or that it combines several policies in a single regulation/accreditation standard. From a methodological perspective, limitations are that the search was conducted in August 2023 and that the search terms focused on PA promotion. An update of the search and/or the use of broader search terms (e.g., related to obesity prevention or health promotion) may have helped to identify additional studies. It also needs to be acknowledged that studies on the effectiveness of organisational policies may provide additional insights to inform evidence‐based political decisions (Adams et al. 2023); however, this type of studies was not covered by the scope of our review. For quality assessment, the adaptation of the Downs and Black (1998) checklist may have limited its sensitivity to different methodological problems in the included studies. In addition, the quality assessment was not conducted at outcome level, i.e., does not differentiate between changes of the PA behaviour and the PA environment.

## 5 Conclusion

The ECEC setting plays a crucial role in promoting PA among preschool children. Public policies have the potential to change the environment and positively influence PA behaviour in preschool children. However, due to the heterogenous methodological approaches in the identified studies, no concluding remarks can be made about the actual effectiveness of policies on the PA behaviour of children in ECEC settings. Despite this, the review was able to identify some positive effects of policies implementing PA into the curriculum, requiring a mandatory PA time of at least 60 min per day or revising accreditation standards on the PA environment. In addition, studies showed a high adherence rate of ECEC centres to binding PA policies; however, this rate was significantly lower when measured objectively. These results indicate that PA policies can have an impact on ECEC facilities and create a PA‐promoting environment for preschool children. However, the limited information on the actual effects of ECEC policies on the PA behaviour highlights the need for further research to investigate the effectiveness of public policies, in particular related to gender‐specific and equity‐related effects of these policies, and in the global south. Consequently, a better understanding of these causal relationships would not only benefit research but could also inform evidence‐based policy making.

## Author Contributions

**Maike Till:** methodology, formal analysis, investigation, data curation, writing – original draft, writing – review and editing, visualization. **Kevin Volf:** investigation, writing – original draft, writing – review and editing. **Clara Tristram:** investigation, writing – review and editing. **Stefanie Do:** writing – review and editing. **Peter Gelius:** funding acquisition, conceptualization, writing – review and editing. **Antje Hebestreit:** funding acquisition, writing – review and editing. **Sylke Oberwöhrmann:** funding acquisition, writing – review and editing. **Sven Messing:** project administration, conceptualization, methodology, writing – original draft, writing – review and editing.

## Conflicts of Interest

The authors declare no conflicts of interest.

## Data Availability

Data sharing is not applicable to this article as no datasets were generated or analysed during the current study.
